# Direct Visualization and Quantitative Insights into the Formation and Phase Evolution of Cu Nanoparticles via In Situ Liquid Phase 4D‐STEM

**DOI:** 10.1002/advs.202500706

**Published:** 2025-03-24

**Authors:** Ningyan Cheng, Hongyu Sun, Yevheniy Pivak, Christian H. Liebscher

**Affiliations:** ^1^ Max‐Planck‐Institut für Eisenforschung Max‐Planck‐Str. 1 40237 Düsseldorf Germany; ^2^ Institutes of Physical Science and Information Technology Anhui University Hefei Anhui 230601 China; ^3^ DENSsolutions B.V. Informaticalaan 12 Delft 2628 ZD The Netherlands; ^4^ Research Center Future Energy Materials and Systems Ruhr University Bochum Universitätsstr. 150 44801 Bochum Germany; ^5^ Faculty of Physics and Astronomy Ruhr University Bochum Universitätsstr. 150 44801 Bochum Germany

**Keywords:** 4D‐STEM, Cu, Electrochemistry, in situ, liquid cell

## Abstract

Copper (Cu)‐based nanomaterials are one of the most efficient heterogeneous electrocatalysts for the CO_2_ reduction reaction. However, their selectivity and stability are strongly determined by their morphology, crystal structure, composition, grain size, grain boundary density, etc. Hence, gaining quantitative insights into their dynamic evolution under synthesis and/or working conditions is critical for developing optimal Cu‐based electrocatalysts and unveiling their structure‐property relationship. In this work, the possibility of addressing these issues is demonstrated by integrating in situ liquid phase transmission electron microscopy (LP‐TEM) with 4D scanning transmission electron microscopy (4D‐STEM). Here, the dynamic morphology and phase evolution of Cu nanoparticles during electrodeposition and electrooxidation processes are revealed in liquid. Virtual imaging and selected area electron diffraction provide novel insights into the evolution of defective nanocrystalline Cu nanoparticles during electrodeposition. It is shown that virtual off‐axis dark field imaging can be used to map the distribution of Cu_2_O and Cu within partially oxidized Cu nanoparticles, opening new opportunities for quantitatively probing electrocatalysts under operando conditions.

## Graphical abstract

By integrating in situ liquid phase transmission electron microscopy (LP‐TEM) with 4D scanning transmission electron microscopy (4D‐STEM), this work not only reveals the morphology and phase evolution of Cu nanoparticles during electrodeposition and electrooxidation processes but also demonstrates the possibility of mapping the grain size and phase distribution of partially oxidized nanocomposites in the liquid.

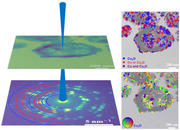

## 1 Introduction

Currently, Cu and its derivates are the most efficient heterogeneous electrocatalysts for electrochemical CO_2_ reduction reaction (CO_2_RR), yet their selectivity and stability still need to be further enhanced.^[^ ^1^ ^]^ It has been demonstrated that the selectivity and stability of catalysts can be tailored by controlling its morphology, crystal structure, composition, grain size, grain boundary density, etc.^[^ ^2^ ^]^ Recent studies also revealed that cube‐shaped Cu‐based nanostructures exhibit higher selectivity toward multicarbon (C2+) products.^[^ ^2^, ^3^ ^]^ Moreover, as the coexistence of Cu^0^ and Cu^+^ can synergistically promote CO dimerization and inhibit C_1_ routes, oxidative‐derived Cu and partially oxidized Cu exhibit higher selectivity toward CO_2_RR than pure Cu and Cu_2_O.^[^ ^2^, ^4^ ^]^ Meanwhile, the distribution of Cu and Cu_2_O also affects the selectivity and activity of the composites.^[^ ^2^, ^4^ ^]^

To achieve a controllable synthesis of desirable Cu‐based electrocatalysts and to understand their structure‐property relationship, a range of in situ/operando techniques such as in situ X‐ray diffraction, in situ X‐ray absorption, in situ transmission electron microscopy (TEM), etc. have been applied to monitor their dynamic evolution under corresponding synthesis and/or working conditions.^[^ ^1^, ^2^, ^3^, ^4^, ^5^, ^6^ ^]^ Among these various techniques, in situ liquid phase TEM (LP‐TEM) stands out as it can reveal the dynamic morphological evolution of nanoscale catalysts not only in liquid‐phase synthesis but also under electrochemical reaction conditions. Nevertheless, the strong electron scattering within thick liquid layers degrades the spatial resolution and makes it challenging to discern dynamic phase, crystal structure, and composition evolution, which are important quantitative parameters for understanding the structure‐property relationship of catalysts and for achieving controlled synthesis conditions.^[^ ^7^ ^]^ For instance, LP‐TEM has been successfully employed to visualize the dynamic electrochemical formation of Cu_2_O and monitor its morphological evolution during subsequent reduction under a reductive potential.^[^ ^3^ ^]^ However, the limited spatial resolution prohibits the extraction of more detailed structural information within the particles. It has been shown that it is possible to acquire in situ selected area electron diffraction (SAED) patterns, but the weak and averaged diffraction signal cannot reveal localized crystallographic information of individual particles. To address these limitations of LP‐TEM, numerous strategies have been proposed, such as expelling the liquid or introducing gases to reduce the liquid layer thickness, mitigating window bulging, etc.^[^ ^8^ ^]^ For example, by inducing gas bubbles through electrochemical water splitting, Serra‐Maia et al. obtained ≈30 nm thick liquid, within which they not only achieved atomic‐scale imaging but also conducted structural and chemical analysis via electron energy loss spectroscopy (EELS) and SAED.^[^ ^8c^ ^]^

Recently, it has been demonstrated that 4D scanning transmission electron microscopy (4D‐STEM) can reveal information on crystal orientations, local strain fields, lattice defects, etc. of various material systems by recording diffraction patterns at each scan position using pixelated detectors/cameras.^[^ ^9^ ^]^ Taking advantage of 4D‐STEM and thin liquid layers obtained by either gas bubbles or a pressure controller system, several studies have revealed the morphology, composition, and structure evolution of selected nanomaterials during corresponding electrochemical reactions.^[^ ^10^ ^]^ For instance, Yang et al. monitored the dynamic evolution of Cu nanoparticles during the electrochemical CO_2_RR process by acquiring the 4D‐STEM data within thin liquid layers formed by H_2_ bubbles.^[^ ^10b,c^ ^]^ It was discovered that under working conditions, these small Cu nanoparticles aggregated to form active nanograins, which subsequently transformed into single‐crystal Cu_2_O nanocubes when exposed to air. However, the behavior observed in thin liquid layers may deviate from that observed in bulk liquid as thin liquid layers exhibit a larger ohmic drop and diffusion as well as mass‐transport restrictions.^[^ ^8^, ^11^ ^]^ This phenomenon is particularly notable in (electro)chemical reactions that are predominantly governed by mass transport. Furthermore, the deliberately introduced gas bubbles not only create additional gas‐liquid interfaces in the reaction system but also alter the chemical and physical properties of the exposed surfaces of the studied materials. For example, the induced gas molecules could diffuse in atomic form into the nanocatalyst along grain boundaries, which are known as diffusion pathways for gas species and active sites for catalytic reactions.^[^ ^2^, ^10^ ^]^ Hence, it is crucial to realize the acquisition of detailed structural and compositional information in LP‐TEM without deliberately reducing the thickness of the liquid.

Here, we observe the dynamic electrodeposition of Cu nanoparticles by STEM imaging and unveil their morphological and crystallographic evolution at different potentials by quasi‐dynamic in situ 4D‐STEM. Virtual bright‐field (BF) and dark‐field (DF) imaging, along with selected area diffraction, reveal that most of the nanoparticles are composed of a nanocrystalline structure and that planar faults exist in some faceted Cu nanoparticles. Subsequently, a dynamic oxidation‐induced hollowing of these nanocrystalline Cu particles was observed during in situ electrooxidation. The corresponding in situ acquired 4D‐STEM data not only reveals the crystal structure of the obtained composite catalyst, but it is even possible to perform phase mapping to obtain the distribution of Cu_2_O and Cu in the composite. By using virtual off‐axis dark‐field imaging, the in‐plane crystal orientations and size distribution of the nanocrystalline Cu_2_O can be determined without changing the liquid layer thickness.

## 2 Results and Discussion

**Figure 1:**
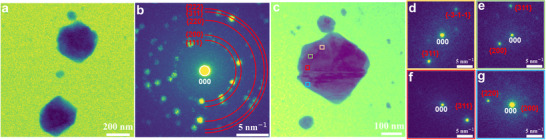
a) STEM image captured at −0.07 V during the electrodeposition process and the b) calculated maximum diffraction pattern derived from the corresponding 4D‐STEM data. c) Zoom‐in of a selected area of the virtual BF image reconstructed by applying a radial virtual aperture around the direct beam as schematically indicated by the white circle in (b). d–g) Mean diffraction patterns obtained from the orange, green, red, and blue squares highlighted in (c).

The in situ electrochemical synthesis of Cu nanoparticles was conducted on a glassy carbon (GC) working electrode within the LP‐TEM cell by subjecting a ≈400 nm thick aqueous solution of 5 mm CuSO_4_ and 5 mm KCl to cyclic voltammetry (CV) (Figures S1–S3, Supporting Information). The dynamic growth process of the particles during the negative sweep was recorded in Video S1 (Supporting Information). Notably, there is a considerable variation in particle size even at the same deposition potential, which possibly stems from the heterogeneous properties of the reference electrodes and the ohmic drop along the working electrode.^[^ ^3^ ^]^ While imaging reveals the morphology and morphological evolution of the nanoparticles, it cannot provide further information about the particles such as composition, crystallography, etc., which are important parameters affecting their catalytic performance. Hence, nanobeam scanning electron diffraction datasets, being a sub‐group of 4D‐STEM techniques, were applied to obtain insights into the local crystallography of the particles. As shown in **Figure** **1**a,b, the maximum diffraction pattern derived from the acquired 4D‐STEM data reveals the particles formed at −0.07 V are metallic Cu, as all the diffraction spots can be assigned to face‐centered cubic (fcc) Cu (Fm‐3 m). Furthermore, the diffraction contrast observed in the virtual BF image (Figure 1c; Figure S4, Supporting Information) reconstructed from the 4D‐STEM dataset indicates the presence of structural defects within these particles. Meanwhile, mean diffraction patterns obtained from several selected regions (Figure 1d–g) unveil the re‐orientation of the crystal lattice within the selected large particle (diameter ≈300 nm), indicating that the particle contains planar faults, such as twins or stacking faults. Such planar faults emerging within the Cu particles may result from their growth process, which could be introduced either by the coalescence of differently oriented Cu particles or during layer growth.^[^ ^12^ ^]^ Notably, directly observing and determining their crystallographic nature can provide novel insights into catalytic reactions as it has been reported that planar fault terminations at the nanoparticle surfaces play an important role in their (electro)catalytic performance.^[^ ^13^ ^]^ Although the particle observed here is in a random crystallographic orientation, which makes it challenging to further characterize it to directly identify these lattice defects without ambiguity, an extension of the method by scanning precession electron diffraction and automated crystal orientation mapping could overcome these limitations even in thick liquid layers.^[^ ^14^ ^]^

**Figure 2:**
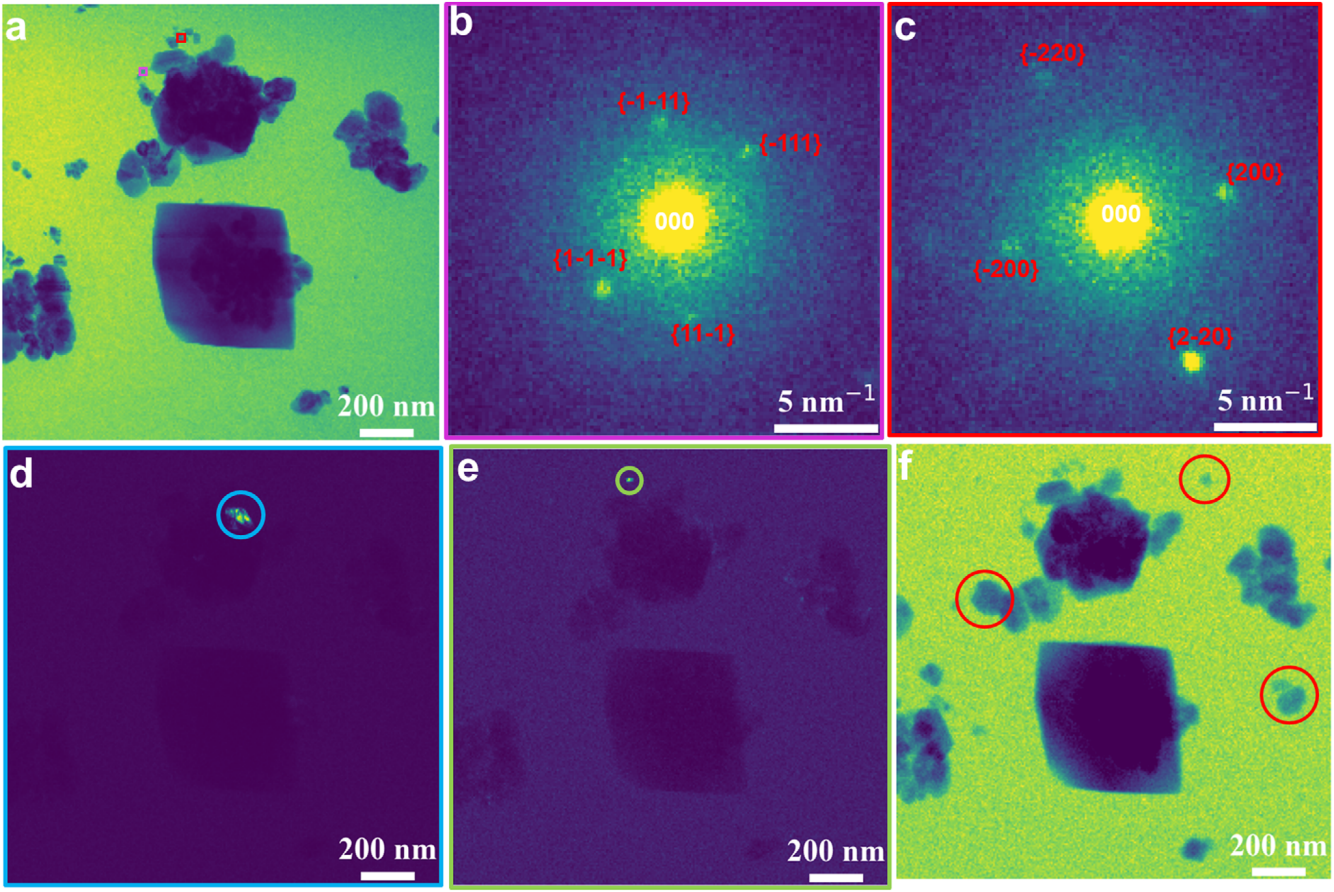
a) Virtual BF image reconstructed using a virtual aperture indicated by a white circle in Figure S5 (Supporting Information). b,c) Mean diffraction patterns of the red and purple squares highlighted in (a), respectively. Virtual off‐axis DF images reconstructed from the d) blue and e) green circles marked in Figure S5 (Supporting Information), respectively. f) STEM image of the same area of (a) captured before acquiring the 4D‐STEM data.

Further inspection of individual diffraction patterns finds that even small electroreduced Cu particles are likely composed of different nanocrystals containing grain boundaries or twins. As shown in **Figure** **2**a–c, the mean diffraction pattern of two randomly selected particles, which are formed at −0.17 V with the size of ≈33 and ≈40 nm in diameter, are extracted from the acquired 4D‐STEM data. It could be seen both of them contain reflections originating from metallic Cu crystals with different orientations, demonstrating both particles are composed of small crystalline grains. Additionally, by selecting specific diffraction spots from the maximum diffraction pattern (Figure S5, Supporting Information), the obtained virtual off‐axis DF images (Figure 2d,e) enable locating different crystals within the complex Cu nanostructure. The reconstructed off‐axis DF images demonstrate that even the diffraction contribution of a ≈28 nm particle (Figure 2e) can be detected for further analysis by 4D‐STEM in liquid. Although, the spatial resolution is limited by the initial probe size and beam broadening in the liquid as well as the SiN*_x_* windows, it enables probing the evolving particle structure and polycrystallinity under electrochemical conditions in a thick liquid layer. Nevertheless, the beam illumination during the acquisition of the 4D‐STEM data did cause slight particle growth, rotation, dissolution, and redeposition of the particles, which can be seen if we compare STEM images taken before and after the 4D‐STEM scan (e.g., compare Figure 2f with Figure S6, Supporting Information). For instance, the particles highlighted by red circles formed at −0.17 V (Figure 2f) seem to have dissolved or migrated out of the field of view during or after the 4D‐STEM experiment (Figure 2a). However, this issue could be mitigated by high‐frame rate 4D‐STEM detectors as it can reduce dose‐related effects and allow acquiring fast dynamic electrochemical processes by in situ 4D‐STEM.^[^ ^15^ ^]^

**Figure 3:**
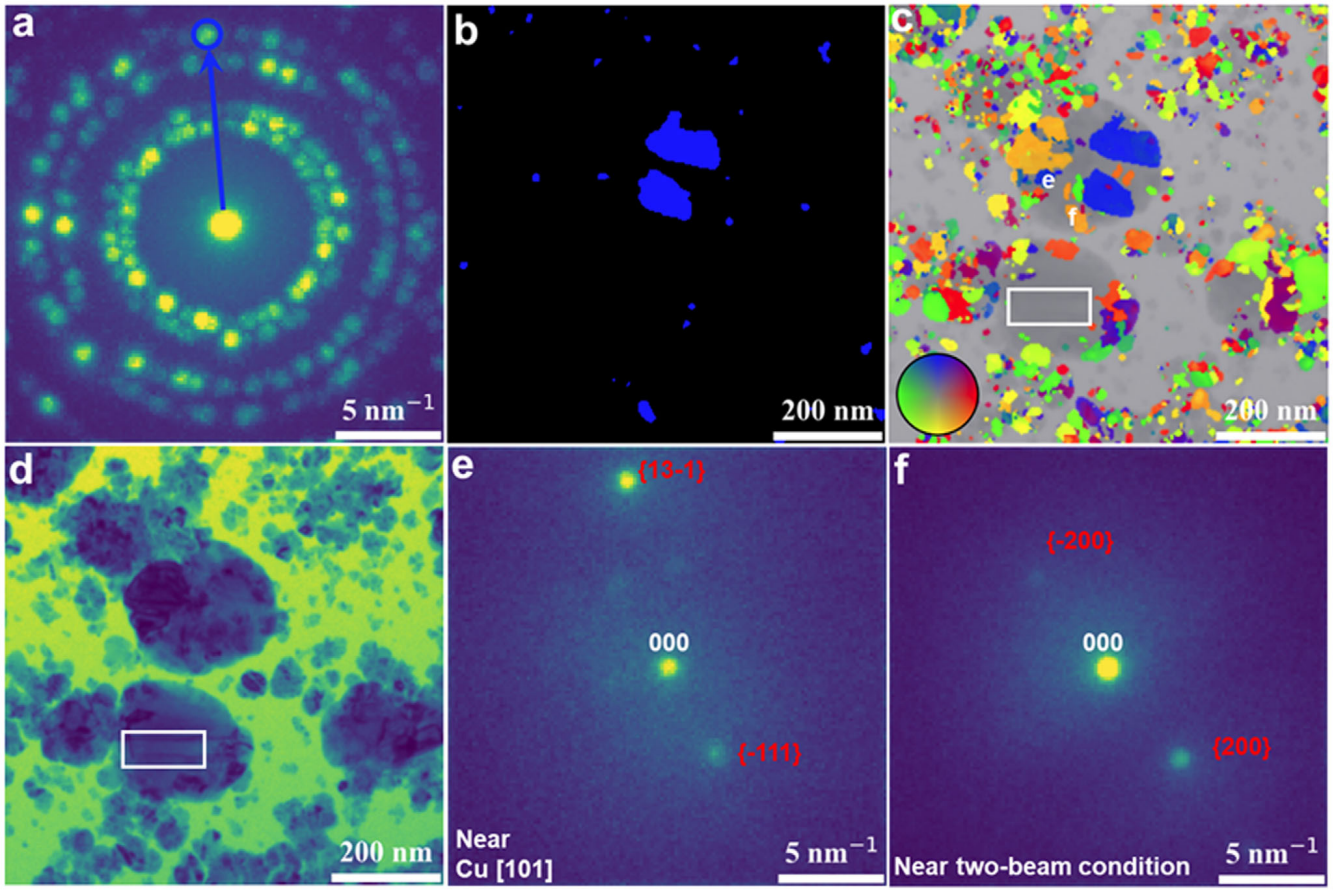
a) Calculated maximum diffraction pattern derived from the 4D‐STEM data acquired at −0.34 V. b) False‐color binarized virtual off‐axis DF image of the selected reflection marked with a blue circle in (a). c) Overlay of the acquired virtual BF image and the corresponding false‐color virtual off‐axis DF map. d) Virtual BF image. e,f) Mean diffraction patterns of the particles labeled as e and f in (c), respectively.

To obtain quantitative information on the distribution of individual crystallites within different Cu particles, we obtained a false colored image by merging different virtual DF images, where the color corresponds to the in‐plane orientation of the selected diffraction vector (**Figure** **3**a–c). A detailed description can be found in the Method section. An example of one binarized and colored off‐axis virtual DF image of the particles formed at −0.34 V is shown in Figure 3b, while the corresponding selected diffraction vector and virtual aperture are illustrated in Figure 3a. As shown in Figure 3c, the false‐color off‐axis DF map is superimposed on the corresponding virtual BF image and provides insights into the nanocrystalline structure and distribution of nanocrystals in different areas of the nanoparticles. Notably, the crystalline information from large crystals (>150 nm in diameter) is missing since the electron scattering signal is too weak to be detected from the background generated by the strong inelastic electron scattering of the thick liquid layer. Nevertheless, it is still possible to obtain information from the inner structure of these particles as can be seen in the virtual BF image reconstructed from the acquired 4D‐STEM data. For instance, while the diffraction signal from the selected white square is difficult to discern from the background and only morphological information can be determined from the STEM image, indications for the presence of planar faults are visible in the corresponding BF image (Figure 3d). Furthermore, detailed information of the distribution of nanocrystals and their orientation can be obtained from the false‐color image, which then enables extracting diffraction information from selected nanocrystals. For example, the mean diffraction pattern of two selected crystals extracted from square regions of ≈17.8 × 17.8 nm^2^ (labeled as **e** and **f** in Figure 3c) shows a strongly exited {13–1} reflection and weaker {−111} in the crystal with label **e**, indicating that this crystal is oriented near to the [101] zone axis (Figure 3e). Meanwhile, the orientation of nanocrystal **f** is close to a two‐beam condition as only a strong reflection from plane {200} alongside a faint reflection from {−200} plane is exited (Figure 3f).

**Figure 4:**
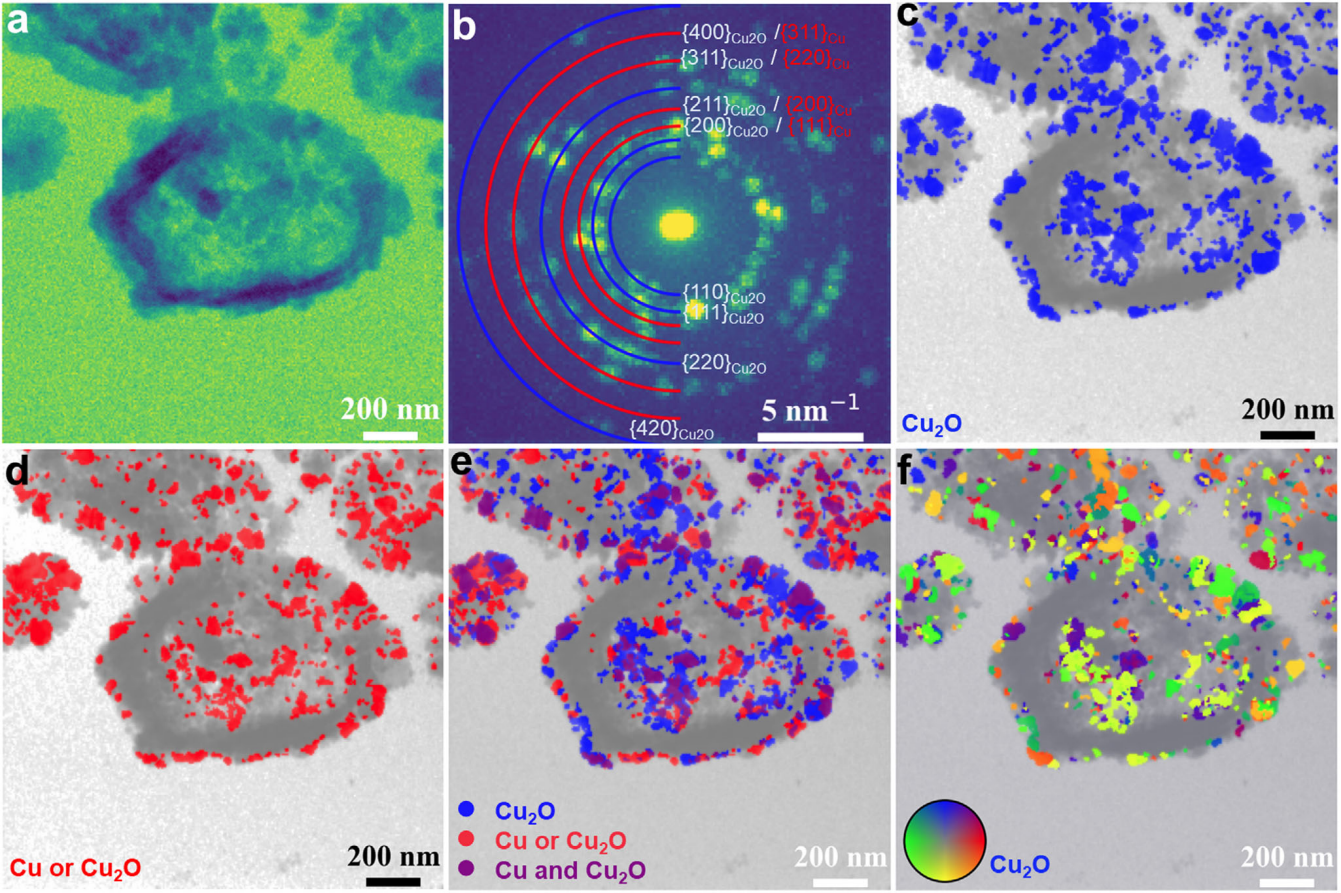
a) STEM image and b) corresponding calculated maximum diffraction pattern of the selected particles shown in (a) after oxidization at OCP. The blue half‐rings indicate characteristic reflections belonging to Cu_2_O, while the red half‐circles indicate diffraction rings that originate from either Cu or Cu_2_O (Cu/Cu_2_O). Overlay of the virtual DF image and the false‐color phase map of c) Cu_2_O and d) Cu/Cu_2_O determined from the diffraction signal of specific Cu_2_O diffraction spots and the ones that could be either Cu or Cu_2_O in (b), respectively. e) Composite map of the selected particles. f) False‐color virtual off‐axis DF map of Cu_2_O.

Given that the selectivity of Cu‐based nanostructures can be adjusted by the ratio of metallic and oxidized Cu and their structure, we monitored the morphological and crystallographic evolution of the newly generated Cu particles during oxidation at open circuit potential (OCP). Video S2 and Figure S7 (Supporting Information) show that OCP induces the nucleation and growth of voids, resulting in hollow particles. Furthermore, there is a correlation between the particle size and its hollowing rate, with smaller particles exhibiting a faster rate of hollowing. To obtain crystallographic information on hollow particles, 4D‐STEM data of a randomly selected relatively large hollow nanoparticle (**Figure** **4**a) was acquired. In the obtained maximum diffraction pattern (Figure 4b), distinct reflections of Cu_2_O (Fm‐3 m) can now be observed, confirming that Cu_2_O has formed during OCP. The other diffraction spots, apart from the distinct reflections of Cu_2_O, can be assigned to either Cu_2_O or Cu as they nearly overlap due to their very close interplanar spacings, which makes it challenging to distinguish them accurately by nanobeam electron diffraction. Nevertheless, these results confirm that Cu can be oxidized under OCP, and the oxidation process results in the hollowing of the nanoparticle via the Kirkendall effect.

The local diffraction information contained in the nanobeam 4D‐STEM dataset can now be used to gain insights into the phase distribution of Cu_2_O in relation to Cu. When using reflections unique to the ordered Cu_2_O structure, it is possible to obtain the distribution of oxide nanocrystals within the hollow particle as shown in Figure 4c. Since the higher order reflections of Cu_2_O overlap with Cu reflections (see Figure 4b), it is only possible to obtain information on where either Cu and/or Cu_2_O has formed when using reflections on diffraction rings with higher scattering angles (as indicated by red labels in Figure 4b), illustrated in Figure 4d. As mentioned before, some areas cannot be identified from the diffraction signal since it is too weak, because the particle regions are too thick or particles/crystals are too small (Figure S8, Supporting Information). To localize where predominantly Cu_2_O has formed and whether Cu and Cu_2_O are overlapping along the beam direction during the hollowing process, the two false‐color phase maps are superimposed. As shown in Figure 4e, the particles which are pure Cu_2_O, Cu/Cu_2_O, and the mixture of Cu_2_O and Cu, are colored blue, red, and dark purple, respectively. It can now be seen that the blue regions are predominantly Cu_2_O crystals, whereas the purple areas can now be identified as an overlap of Cu and Cu_2_O, where either Cu is not fully oxidized or Cu_2_O has grown on top or in between Cu crystals. When looking into the false‐color virtual off‐axis DF map in Figure 4f of Cu_2_O, one can readily see its nanocrystalline structure. From individual binarized images, it is possible to determine the crystal size distribution (Figure S9, Supporting Information), where it can be seen that most of the Cu_2_O crystals are less than 36 nm in diameter, with a peak at 16 nm in diameter (8 nm radius). The largest crystals exhibit a diameter of more than 80 nm.

The above results demonstrate the possibility to map the composition distribution, as well as the grain size, of partially oxidized composites in the liquid. This data is key to understanding the relationship between the structure and properties of nanocatalysts, as their oxidation state, grain boundary characteristics, and phase distribution strongly influence their (electro)catalytic performance.^[^ ^16^ ^]^ However, significant inelastic scattering primarily in the thick liquid layer limits the detection of weak diffraction signals and thus hinders the crystal or phase identification of small and large objects. To reduce inelastic scattering contributions, energy‐filtered 4D‐STEM could be applied in future studies. In addition, scanning precession electron diffraction in conjunction with automated crystal orientation mapping could be used to determine the character of the grain boundaries and grain boundary surface terminations during the oxidation step to determine the role of the grain boundaries on Cu_2_O formation. Moreover, they could also be used to improve the accuracy of grain size determination as the dynamic diffraction effect could be mitigated.

## 3 Conclusion

Taking advantage of in situ LP‐TEM and 4D‐STEM techniques, we monitored the dynamic morphological and crystallographic evolution of Cu nanoparticles during potential‐driven generation and oxidation in a ≈400 nm thick electrolyte layer. This work shows that by using virtual BF imaging and selected area diffraction, indications for the formation of planar faults within Cu nanoparticles can be resolved, which are otherwise cannot be observed by STEM imaging in thick liquid. Moreover, it is demonstrated that local diffraction information can even be obtained of nanoparticles with less than 30 nm in diameter and that they can be localized by virtual DF imaging. Quantitative crystal orientation/phase mapping was applied to reveal the crystal orientation and to monitor the oxidation and phase evolution of the Cu nanostructures. The results demonstrate that it is possible to unveil the distribution of Cu_2_O and Cu, as well as their grain size distribution, in composite nanoparticles after partial oxidation. This information is crucial in correlating the structure and properties of nanocatalysts as it has been shown that their oxidation state and (electro)catalytic performance are highly affected by grain boundary density, grain boundary sites, grain size, and phase distribution. Thus, we believe this work provides new pathways for combining in situ LP‐TEM and 4D‐STEM to provide quantitative insights into the structure and phase evolution of electrocatalysts required for uncovering their structure‐property relationship.

## 4 Experimental Section

Copper (II) sulfate (CuSO_4_) and potassium chloride (KCl) were purchased from Aladdin Chemical Co.Ltd. All of the chemicals were used as received without any further purification. The water used throughout the experiments was deionized water.

### Methods

To enable in situ LC‐STEM investigations, a so‐called nano‐cell was assembled, consisting of a top and a bottom chip, each featuring a central viewing window made of 50 nm thick SiN*_x_*. The bottom chip, as illustrated in Figure S1 (Supporting Information), comprises a counter electrode (CE), a reference electrode (RE), and working electrode (WE). The WE was fabricated by glassy carbon (GC) because of the electrochemical inert nature and low contrast for imaging, and both the RE and CE are made of Pt due to the chemical stability. To prevent the possible interfacial side reactions from occurring outside the window area, all the electrodes were encapsulated with SiN*_x_* except for the electrode area inside the microfluidic path. The bottom chip also has an integrated liquid inlet, a flow channel, and an outlet. The windows of the two chips are parallel with each other, which was beneficial for observations in a large area.

The Nano‐Cell was assembled on the Stream LB holder, which was then subject to the leakage test in a pumping station. The leak‐tight holder was inserted into a Thermo Fisher Scientific Titan Themis microscope at 300 kV equipped with a probe corrector, a four‐segment annular detector, a Gatan Quantum Imaging Filter with dual‐electron energy‐loss spectroscopy (EELS) system, and an electron microscope pixel array detector (EMPAD). After imaging the dry window and the WE, the holder was reconnected to a liquid flow system via a polyetheretherketone (PEEK) tubing set. The electrolyte, a mixture of 5 mM CuSO_4_ and 5 mM KCl aqueous solution, was driven from the inlet through the field of view and the outlet by gas pressure pumps. The liquid flow rate can be controlled by independently changing the inlet and outlet pressure of the Nano‐Cell. STEM‐EELS data was acquired in STEM mode with a dispersion of 0.05 eV per channel and a pixel acquisition time of 0.1 s using the Quantum GIF while the convergent semi‐angle of the beam was 17.0 mrad and the collection semi‐angle was 6.8 mrad. EELS measurements indicate the assembled dry cell exhibits a uniform thickness of ≈100 nm, as each of the two window membranes was designed to be ≈50 nm thick (Figure S2, Supporting Information). Once the cell was filled with the mixed electrolyte of CuSO_4_ and KCl, the liquid thickness increased continuously from the corners to the center (Figure S2, Supporting Information). The liquid thickness was ≈200 nm in the corners of the cell, while in the center, it was ≈400 nm. This observed variation was consistent with prior reports and results from membrane bulging induced by the pressure difference between the TEM column and the enclosed liquid cell.^[^ ^8b^ ^]^

A PalmSens 4 potentiostat was used for electrochemical control. Cyclic voltammetry (CV) was performed by applying a potential ranging from −0.7 to 0.2 V versus Pt, scanning from 0 to 0.2 then from 0.2 to −0.7 V at a sweep rate of 100 mV s^−1^. The 4D‐STEM data were acquired by an EMPAD with a converged nanobeam using a convergence semi‐angle of 0.46 mrad, a probe scan grid of 256 × 256 pixels, and a pixel dwell time of ≈1 ms. To guarantee sufficient signal‐to‐noise ratio and to mitigate electron beam‐related effects in the liquid, dose rates of ≈7–14 e Å^−2^ s^−1^ were used. The 4D‐STEM data was processed using the open‐source toolbox py4DSTEM^[^ ^17^ ^]^ and custom‐written workflows mainly employing thresholding and image filters implemented in scikit‐image. To generate the false‐color virtual off‐axis DF maps, first, virtual off‐axis DF images for the corresponding diffraction rings of Cu and Cu_2_O were automatically extracted using a radius of the virtual aperture of 3 pixels. Each of the virtual DF images was then binarized by thresholding with the triangle method followed by smoothing using a median filter. The binarized images were then colored according to the in‐plane direction or angle of the chosen scattering vector of the virtual dark‐field aperture. The final color‐coded image and the color wheel representing the scattering vectors of the virtual apertures are obtained by EMPyRE.^[^ ^18^ ^]^ Due to the significant inelastic scattering introduced by the thick electrolyte within the cell and the inherently weak electron scattering signals from both small and large crystals, only a limited number of diffraction spots can be distinguished from the background in the mean diffraction pattern of selected areas. As a result, we can only assign the spots to the specific family of planes, but not to the specific plane. Therefore, curly brackets were used to label the diffraction spots in the mean diffraction patterns instead of parentheses.

## Conflict of Interest

The authors declare no conflict of interest.

## Supporting information



Supporting Information

Supplemental Video 1

Supplemental Video 2

## Data Availability

The data that support the findings of this study are available from the corresponding author upon reasonable request.
