# Supplementary material for: Direct Visualization and Quantitative Insights into the Formation and Phase Evolution of Cu Nanoparticles via In Situ Liquid Phase 4D‐STEM
Source: Adv Sci (Weinh). 2025 Mar 24;12(19):2500706. doi: 10.1002/advs.202500706 (PMC12097071; doi:10.1002/advs.202500706)
Supplement: Supplementary file 1 — Supporting Information [file ADVS-12-2500706-s001.docx]

**Supporting Information**

**Direct Visualization and Quantitative Insights into the Formation and Phase Evolution of Cu Nanoparticles via In Situ Liquid Phase 4D-STEM**

Ningyan Cheng,* ^[a,b]^ Hongyu Sun,^[c]^ Yevheniy Pivak,^[c]^ and Christian H. Liebscher*^[a,d,e]^

[a] Dr. N. Cheng, Prof. C. H. Liebscher

Max-Planck-Institut für Eisenforschung
Max-Planck-Str. 1 40237 Düsseldorf, Germany
E-mail: [n.cheng@mpie.de](mailto:n.cheng@mpie.de); [christian.liebscher@rub.de](mailto:christian.liebscher@rub.de)

[b] Dr. N. Cheng
Institutes of Physical Science and Information Technology
Anhui University
Hefei 230601, Anhui, China

[c] Dr. H. Sun, Dr. Y. Pivak
DENSsolutions B.V.

Informaticalaan 12, 2628 ZD Delft, The Netherlands

[d] Prof. C. H. Liebscher

Research Center Future Energy Materials and Systems

Ruhr University, Bochum

Universitätsstr. 150, 44801 Bochum, Germany

[e] Prof. C. H. Liebscher

Faculty of Physics and Astronomy

Ruhr University, Bochum

Universitätsstr. 150, 44801 Bochum, Germany


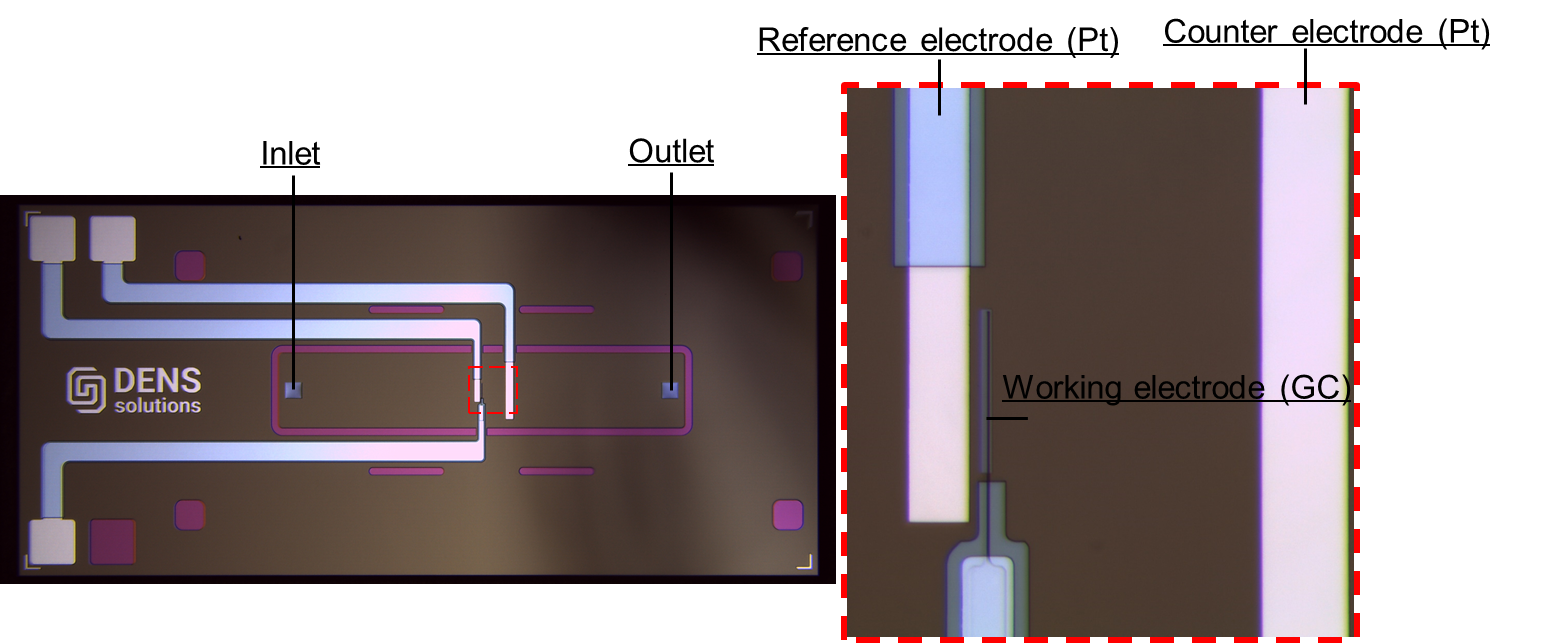


**Figure S1** The geometry and three-electrode configuration on the bottom chip for in-situ electrochemical TEM studies. The WE is fabricated from glassy carbon while the RE and CE are made of Pt. The three electrodes are parallel to each other and perpendicular to the liquid flow direction. Given that the liquid flow direction is perpendicular to the long side of the GC working electrode, the generation of dendrites on one of the long edges of the GC electrode during the growth of Cu (as shown in **video S1**) possibly results from the elevated electrolyte concentration near this particular edge.


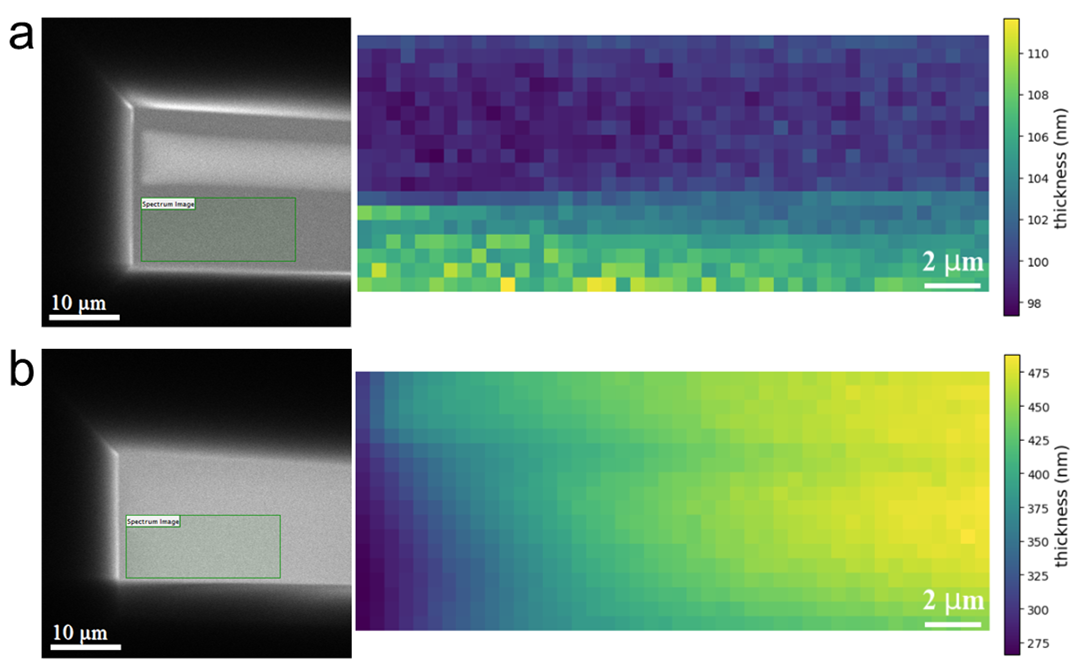
**Figure S2** STEM images and the thickness maps of a selected area of the sealed cell in (a) dry and (b) liquid-filled states, respectively.


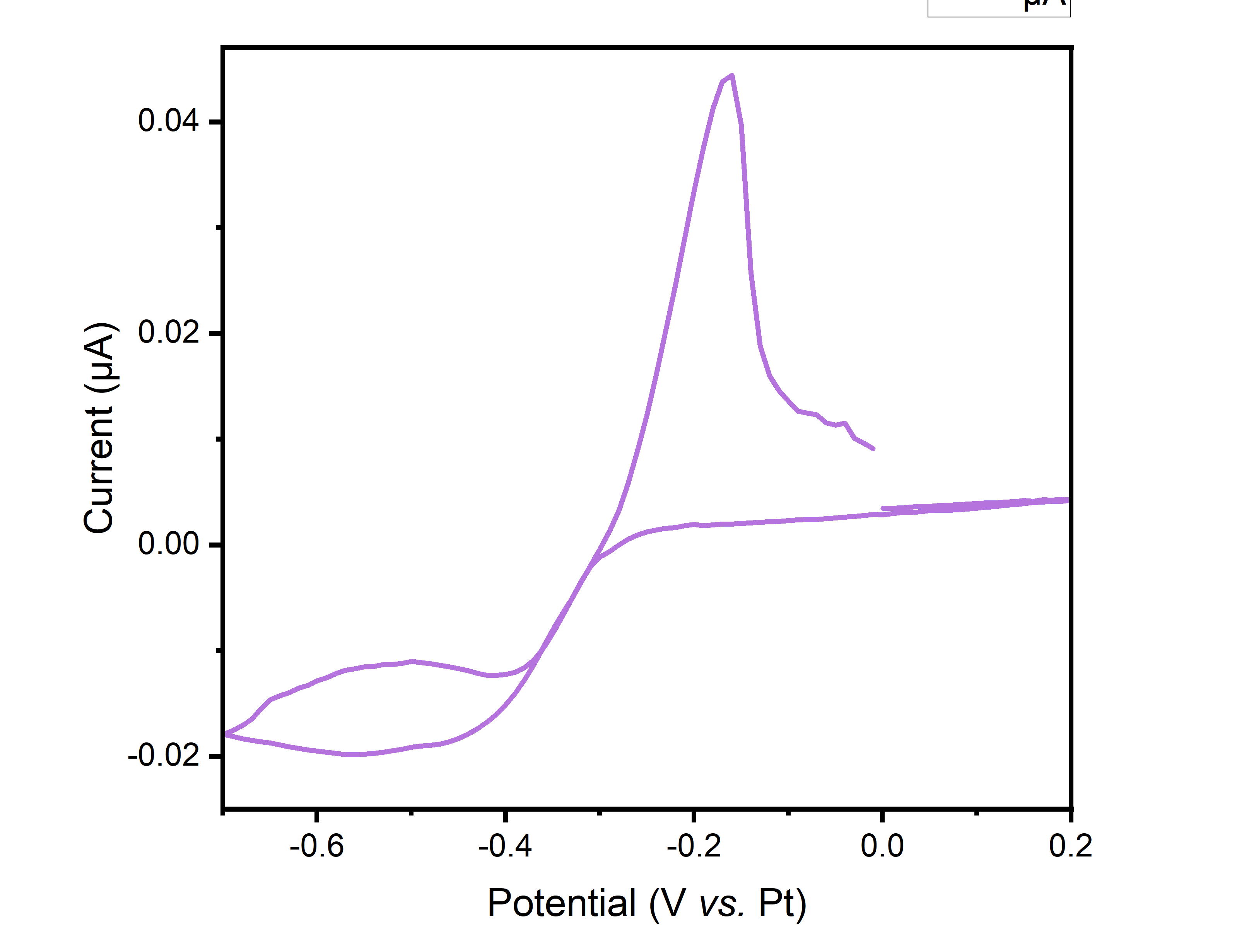


**Figure S3** A typical cyclic voltammetry curve recorded at a scan rate of 100 mV/s in a mixed electrolyte containing 5 mM CuSO_4_ and 5 mM KCl solution.


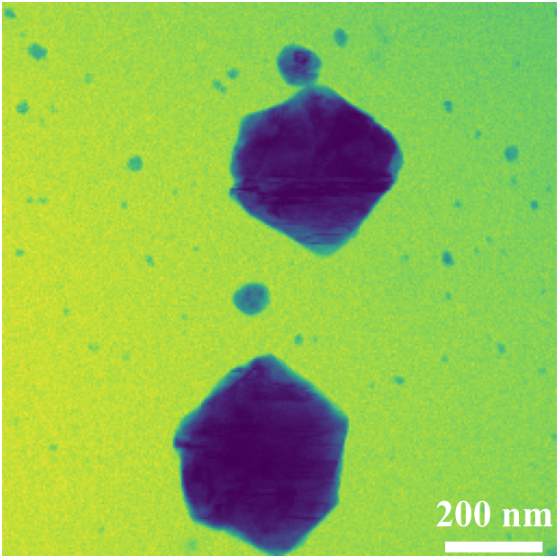


**Figure S4** Virtual BF image reconstructed by applying a radial virtual aperture around the direct beam as schematically indicated by the white circle in Fig. 1b.


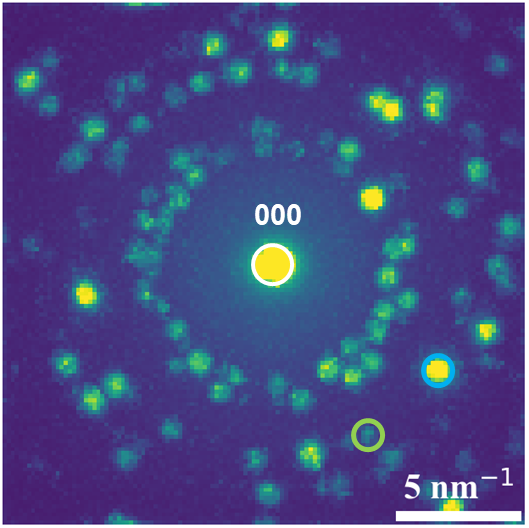


**Figure S5** Maximum diffraction pattern obtained from the 4D-STEM data of the particles shown in Fig. 2a.


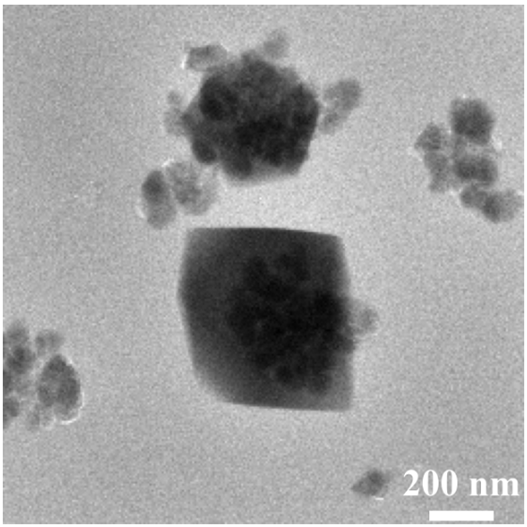


**Figure S6** STEM image of the same area of Fig. 2f captured after acquiring the 4D-STEM data shown in Fig.2.


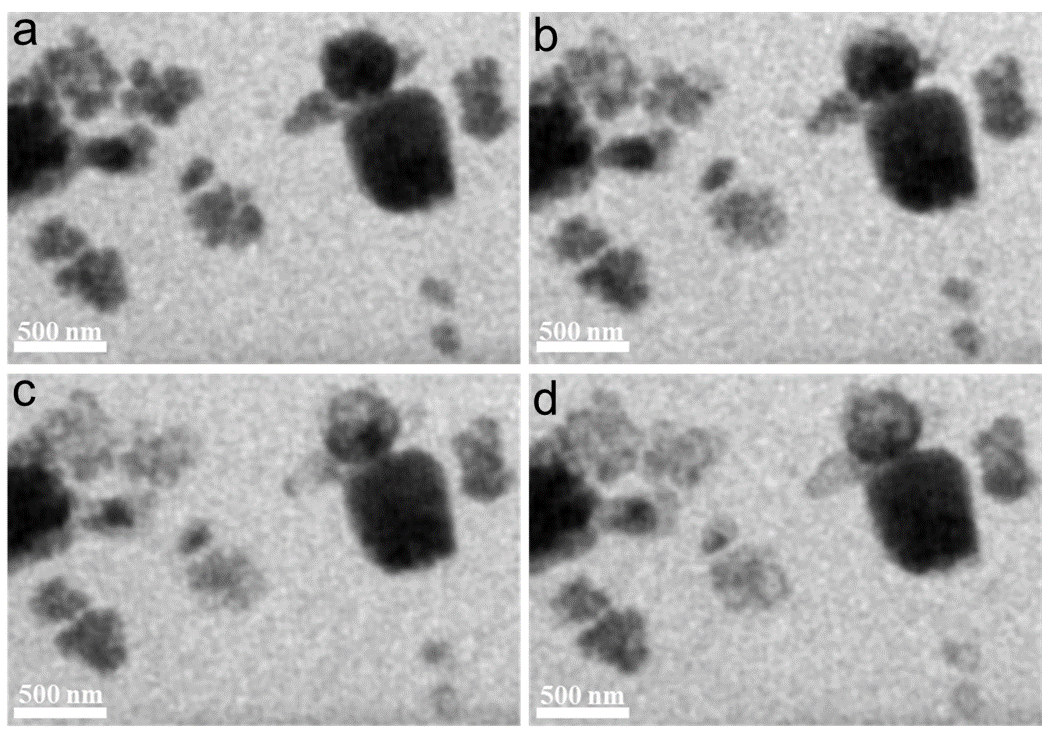
**Figure S7** Snapshots from the in situ video (**Video S2**) which was acquired while OCP was applied to the newly formed Cu particles.


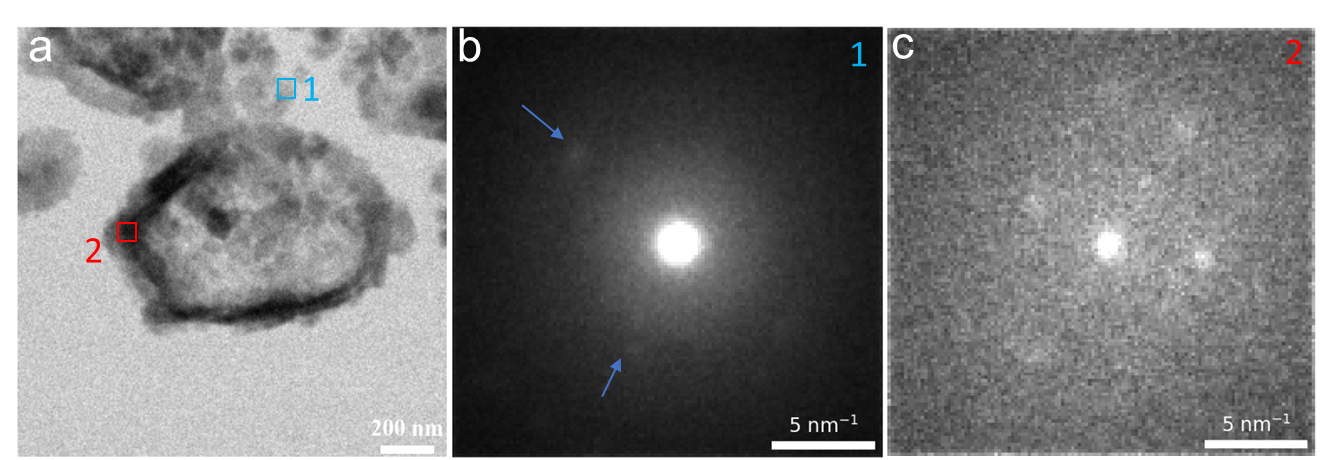


**Figure S8** (a) STEM image of the selected particles obtained after holding at OCP. **(**b, c**)** Mean diffraction pattern of the two selected areas marked in (a), respectively.


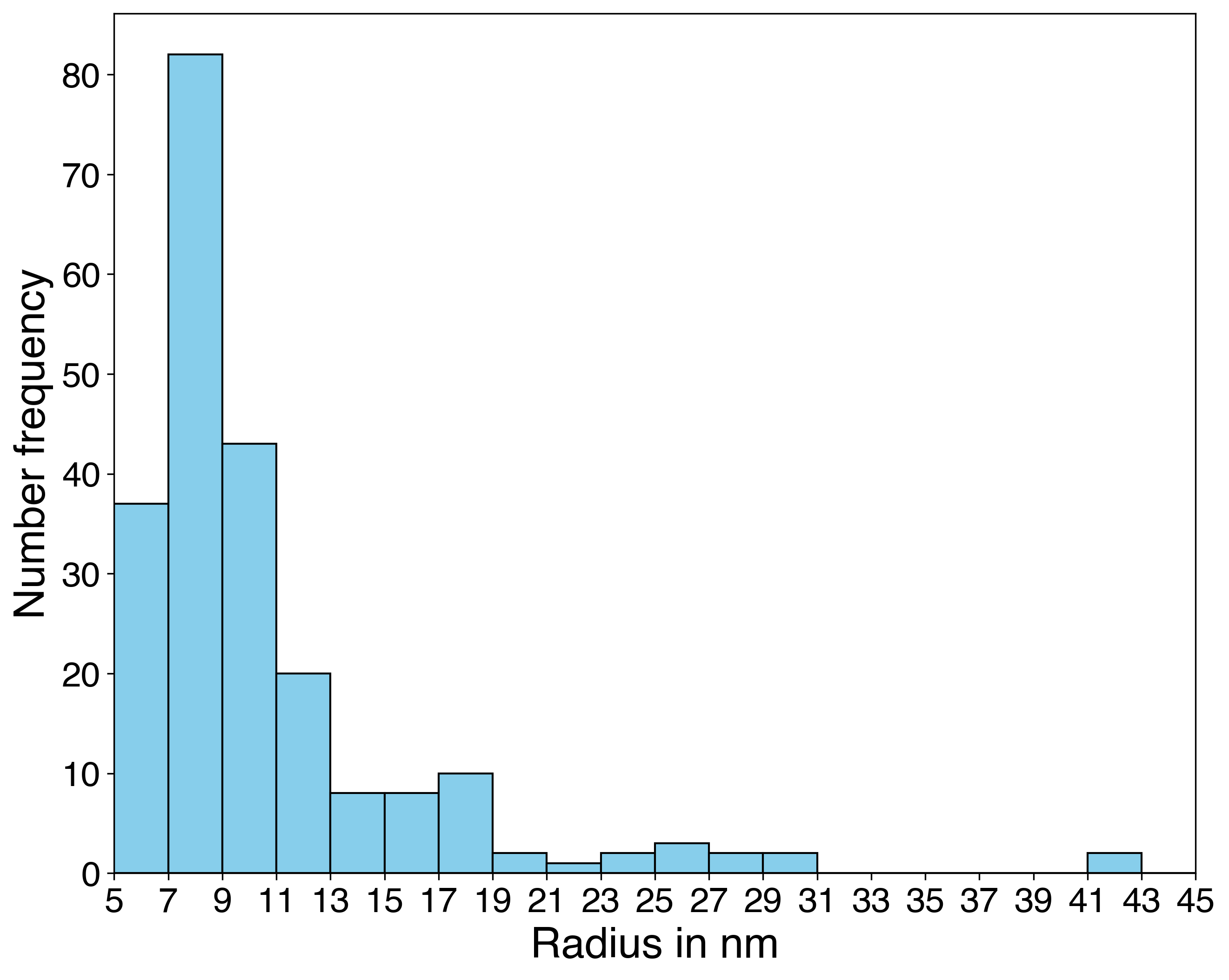


**Figure S9** Crystal size distribution derived from the false-color virtual off-axis DF map of Cu_2_O in Fig.4f.
